# A retrospective analysis of hand tapping as a longitudinal marker of disease progression in Huntington’s disease

**DOI:** 10.1186/1471-2377-14-35

**Published:** 2014-02-24

**Authors:** Lucy M Collins, Stanley E Lazic, Roger A Barker

**Affiliations:** 1 John van Geest Centre for Brain Repair, Cambridge, UK; 2 In Silico Lead Discovery, Novartis Institutes for Biomedical Research, Basel, Switzerland

**Keywords:** Huntington’s disease, Biomarker, Hand tapping

## Abstract

**Background:**

Current clinical assessments of motor function in Huntington’s Disease (HD) rely on subjective ratings such as the Unified Huntington’s Disease Rating scale (UHDRS). The ability to track disease progression using simple, objective, inexpensive, and robust measures would be beneficial.

**Methods:**

One objective measure of motor performance is hand-tapping. Over the last 14 years we have routinely collected, using a simple device, the number of taps made by the right and left hand over 30 seconds in HD patients attending our NHS clinics.

**Results:**

Here we report on a longitudinal cohort of 237 patients, which includes patients at all stages of the disease on a wide range of drug therapies. Hand tapping in these patients declines linearly at a rate of 5.1 taps per year (p < 0.0001; 95% CI = 3.8 to 6.3 taps), and for each additional year of age patients could perform 0.9 fewer taps (main effect of age: p = 0.0007; 95% CI = 0.4 to 1.4). Individual trajectories can vary widely around this average rate of decline, and much of this variation could be attributed to CAG repeat length. Genotype information was available for a subset of 151 patients, and for each additional repeat, patients could perform 5.6 fewer taps (p < 0.0001; 95% CI = 3.3 to 8.0 taps), and progressed at a faster rate of 0.45 fewer taps per year (CAG by time interaction: p = 0.008; 95% CI = 0.12 to 0.78 taps). In addition, for each unit decrease in Total Functional Capacity (TFC) within individuals, the number of taps decreased by 6.3 (95% CI = 5.4 to 7.1, p < 0.0001).

**Conclusions:**

Hand tapping is a simple, robust, and reliable marker of disease progression. As such, this simple motor task could be a useful tool by which to assess disease progression as well therapies designed to slow it down.

## Background

Huntington’s disease (HD) is an autosomal dominant neurodegenerative disorder that is caused by the expression of mutant huntingtin secondary to a polyglutamine (CAG) expansion in exon 1 of the huntingtin gene [1]. The disease is characterised by the dysfunction, and then loss, of specific neuronal populations especially in the striatum as well as in the cerebellar cortex, thalamus, cerebral cortex and hippocampus [2,3] in association with early posterior white matter changes [3-5]. Typically patients present in mid life with an array of motor signs including chorea and bradykinesia as well as psychiatric and cognitive impairments [6]. Many studies have sought to identify the earliest changes in HD and subtle impairments in motor and cognitive function before predicted disease onset have been reported [7,8]. Others have sought to more objectively track disease progression once the disease has become manifest and this includes a range of motor, cognitive and imaging approaches [4,9]. Such objective markers are increasingly needed as we move towards a time when disease modifying therapies for HD are coming to trial [10].

Currently the gold standard for looking at motor impairments in HD is the Unified Huntington’s Disease Rating Scale (UHDRS), which was primarily designed for manifest HD [11]. The UHDRS however, is susceptible to subjective error and inter-rater variability and has limited sensitivity in early HD patients [12]. We, and others, have therefore sought to find simple motor measures which may be accurately used for tracking disease. In the TRACK-HD baseline analysis, voluntary self paced finger tapping was shown to be a sensitive task in premanifest patients and is associated with disease burden in HD patients [4]. Speed finger tapping tasks used in cross-sectional studies has also been shown to be a sensitive early marker of change in premanifest and manifest HD, with the deficits being more pronounced in later stages of HD [13]. These late change deficits also correlate with atrophy on MRI and clinical scores in HD patients thereby linking structure to function [13]. In another cross-sectional study HD patients had larger variability in hand and finger tapping rates compared with controls and this correlated with cognitive impairments in these patients [14]. In a pre-diagnostic study changes in button tapping showed a significant change in rate of decline as the subjects neared disease onset [13]. In the Predict-HD study, premanifest HD patients also showed variability in speed and self paced tapping although this needs to be validated with longitudinal follow up [15]. Collectively, the data from the TRACK-HD study showed that tapping speed was a robust measure in premanifest and HD patients at 12, 24, and 36 months [5,9,16]. Premanifest patients also showed a decrease in the number of taps performed in the Predict-HD study at 2 years follow up [17]. Our group has previously shown in a 10 year follow up study that the rate of decline in hand tapping correlated with UHDRS motor scores [18].

In addition to hand and finger tapping, other motor measures have been found to be sensitive markers of disease progression including grip force and tongue protrusion tasks. Similar to tapping, performance on these tasks also deteriorates over time in both premanifest patients close to disease onset and in manifest patients in comparison to controls [5,9,17]. The oculomotor system has also been studied in HD and correlates with other motor features [19], even in longitudinal follow up [20]. In particular, changes in saccades such as increased error rates, saccade latency and increased variability of saccade latency has been shown in premanifest and HD patients, with increasing abnormalities in advanced HD patients [19]. Over a three year period premanifest and HD patients displayed significantly increased saccade latencies compared to controls, which could be used as a predictor of time to disease onset in premanifest patients [20].

Finally, attempts have been made to look at more complex motor tasks such as the use of the peg board test, which measures the time taken to insert 25 pegs from a rack into a series of appropriate holes [21]. It has been shown that patients are impaired in this task, although there does not appear to be any significant difference in patients in the premanifest versus manifest stages of the illness [21,22]. When testing complex tasks, execution speed of rapid alternating motion sequences are slower in HD patients compared to controls [23].

In the analysis reported in this paper, we have used a simple hand tapping task, which we have previously shown to be useful in a small cohort of patients followed over time [18]. We now report on the utility of this test in a much larger numbers of patients followed over a longer period of time (up to 14 years), to show that this single measure gives a robust annual decline irrespective of disease stage and therefore could be used as one of an array of assessment tools in trials in manifest HD.

## Methods

### Patients

237 patients were recruited from the regional NHS HD clinic at the Cambridge Centre for Brain Repair between 1998 and 2012, and hand tapping data was collected as part of their routine clinical assessment. Patients were seen typically at intervals of approximately 6 or 12 months, although some were seen more frequently for reasons of clinical care and management. All patients included in the analysis were seen for at least one year and for at least three visits and all were taking their normal medication for their HD. There were no inclusion or exclusion criteria outside of this, and thus and the patients are a representative sample of patients with HD. This analysis was classified as a service evaluation and registered with the Patient Safety Unit at Addenbrooke’s Hospital (Project Register Number: 3026). Since hand tapping data was not initially collected for research purposes and the analysis used fully anonymised data, ethics review was not required and informed consent was not obtained. All patients had a positive genetic test for HD or had features of the disease and came from a known HD family and were diagnosed as having manifest disease when they had a diagnostic confidence level score of >3. The calculated long allele CAG length for the patients in our cohort ranged from 37 to 62 repeats, this was calculated by PCR analysis as previously described [24]. Many of the patients had their genetic test before 2001 when the exact repeat number was not routinely recorded. In those where a CAG length was known (n = 151), their demographic information is summarised in Table 1. The patients overall total functional capacity (TFC) [25,26] was assessed using the UHDRS which ranges from 0–13, with 13 equating to normal independent life and 0 to a total dependence on others. The motor UHDRS score was calculated at the patients first visit and ranged from 0–86. Higher scores indicate a more severe motor impairment [11].

**Table 1:** Demographic information at first visit

	**All patients**	**With CAG information**		
	**(Male = 115, Female = 122)**	**(Male = 71, Female = 80)**		
	**Mean (SD)**	**Range**	**Mean (SD)**	**Range**
**Age**				
Male	49.1 (13.7)	14.0 – 75.6	47.3 (13.5)	14.1 – 75.6
Female	50.0 (12.8)	18.5 – 77.9	49.3 (12.9)	18.8 – 77.9
**Follow up (years)**				
Male	4.6 (2.6)	1.0 – 12.9	4.7 (2.3)	1.3 – 10.6
Female	4.9 (2.5)	1.0 – 14.1	4.5 (2.3)	1.0 – 14.1
**Number of visits**				
Male	7.1 (4.9)	3 – 25	6.8 (4.2)	3 – 22
Female	6.6 (4.2)	3 – 26	6.6 (3.5)	3 – 26
**TFC**				
Male	9.5 (3.4)	2 – 13	10.4 (2.9)	3 – 13
Female	9.1 (3.4)	3 – 13	9.5 (3.3)	3 – 13
**UHDRS motor score**				
Male	23.3 (17.1)	0 – 86	20.2 (13.6)	0 – 51
Female	25.8 (18.4)	0 – 70	23.3 (17.5)	0 – 62
**Disease duration (years)**				
Male	5.3 (3.3)	0.1 – 17.0	4.4 (2.4)	0.1 – 10.5
Female	5.4 (3.2)	0.2 – 15.8	4.7 (2.4)	0.4 – 14.2
**CAG length**				
Male			44.6 (5.1)	39 – 62
Female			43.4 (3.9)	37 – 61

Information is provided separately for the complete data set (N = 237) and for patients with information on CAG repeat length (N = 151).

### Hand tapping device

The original hand-tapping device was designed as a simple objective measure of motor function for use in the routine assessment of patients in clinic. It consists of two buttons 6 cm in diameter, mounted with their centres 30 cm apart. The subject is asked to alternately tap one button after the other as rapidly as possible using their right hand for 30 seconds, and then again with their left hand. The total number of taps for each hand is recorded manually and then summed to give a total number of taps for both hands. This device is different to that used previously by us, which was a more sophisticated device that automatically downloaded data onto a computer and calculated inter tap intervals [18]. Hence, the device used in the present study only allowed us to collect data on the total number of taps.

### Statistical analysis

The main outcome for all analyses was the total number of taps, which was the sum of the number of taps made with the left and right hand. Even though the data are counts (non-negative integers), the data distribution was approximated well by a normal model (the values were far away from zero), which was therefore used instead of a Poisson or negative binomial model. The first analysis used all 237 patients and estimated the change in number of taps over time with a mixed-effects model. Fixed factors were time (since first visit), age at first visit, sex, and time by sex interaction. The random factors were patient and patient by time interaction (varying intercepts and varying slopes). The serial dependence of observations within patients was modeled using an exponential correlation structure. The second analysis used the 151 patients for which information on CAG length was available. The same mixed-effects model as the first analysis was used with the addition of CAG length as a continuous variable and the removal of the non-significant time by sex interaction.

Finally, the relationship between tapping and TFC was examined with a mixed-effects model using the 227 patients for which TFC data was available. The model included a fixed effect of age, sex, and TFC, and random effects for patient and patient by TFC interaction. Analysis was conducted with R (3.0.0).

## Results

### Change in hand tapping over time for all patients

**Figure 1:**
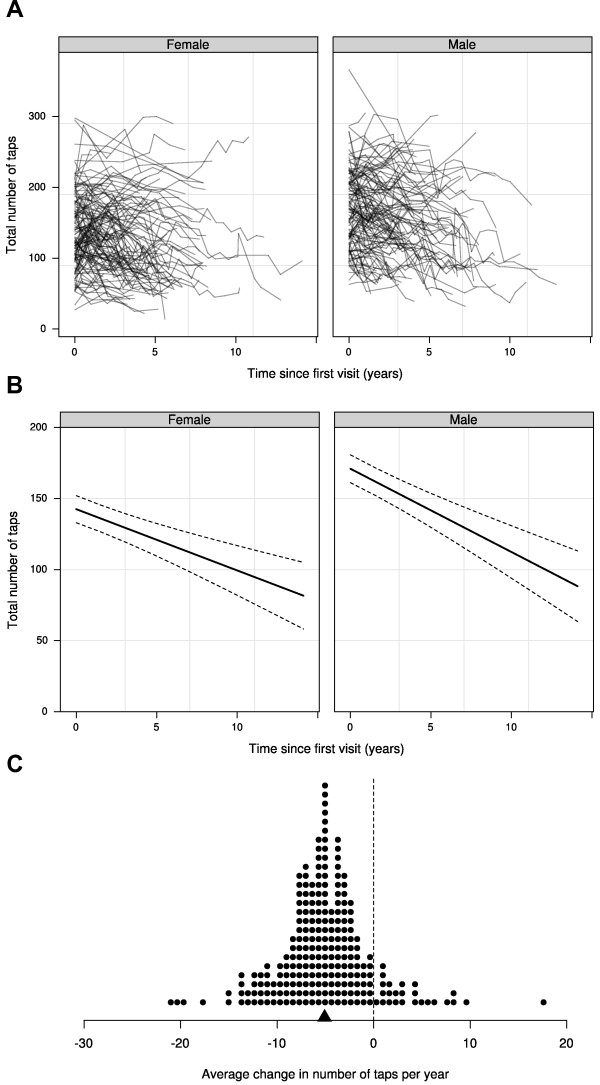
**Change in hand tapping over time by sex.** Individual profiles for 237 patients **(A)** and the population averages with 95% CI (dashed lines; **B**). On average, patients performed 5.1 fewer taps each year (p < 0.0001), but there were large differences in hand tapping scores between patients. This can be seen in panel **(C)**, which shows the estimated annual change for each individual. The population mean is indicated with the triangle on the x-axis.

The number of taps declined linearly over time; patients performed 5.1 fewer taps each year (main effect of time: p < 0.0001; 95% CI = 3.8 to 6.3 taps; Figure 1) with no difference between sexes in the rate of decline (no time by sex interaction: p = 0.207; Figure 1B), although females on average performed approximately 28 fewer total taps than males (main effect of sex; p = 0.0001). The estimates for each individual are shown in Figure 1C and it should be noted that the individuals in the tails of the distribution were not necessarily the ones with the fewest observations or the shortest follow up time. Older patients performed fewer taps than younger patients; so for each additional year of age, patients performed 0.9 fewer taps (main effect of age: p = 0.0007; 95% CI = 0.4 to 1.4). Nevertheless, if we assume that patients would have decreased at a rate of 0.9 taps per year in the absence of the disease, then approximately 82% of the annual decrease can be attributed to the effect of the disease ([(5.1 - 0.9)/ 5.1] * 100 = 82%).

### A greater number of CAG repeats is associated with a faster rate of decline in hand tapping

**Figure 2:**
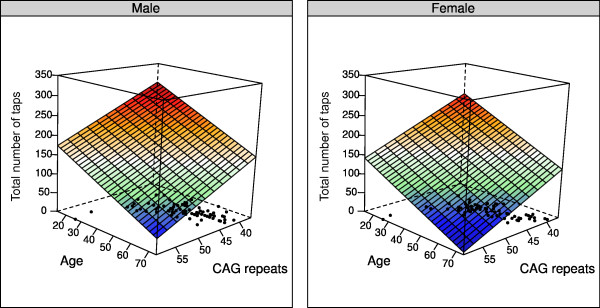
**Hand tapping as a function of CAG length, age, and sex at baseline (first visit).** Older patients and those with a greater number of repeats perform fewer taps. In addition, females perform fewer overall taps than males (p = 0.0001; female plane is shifted downward). Black points on the floor are the locations of the observed data values for age and CAG length. As can be seen, there are no old patients with a high number of repeats.

**Figure 3:**
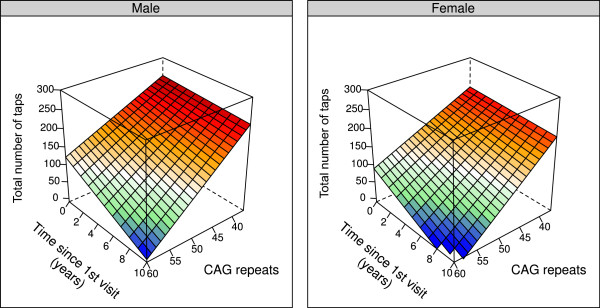
**Change in hand tapping over time by CAG length and sex.** The rate of decline is faster in patients with a greater number of repeats. This can be seen by the steeper slope in the time direction when CAG length = 60 compared to when CAG length = 37, and represents the time by CAG length interaction (p = 0.008). The female plane is parallel to the male but shifted downward, indicating that females perform fewer taps overall. Age at first visit was held constant at 35 years in this figure, and differences in the number of taps at time = 0 reflect the effect of different CAG lengths (e.g. a male with CAG length = 60 who first visits the clinical at age 35 would perform approximately 125 taps, whereas with CAG length = 37 he would perform approximately 250 taps). Graphs using a different age would simply shift the planes up or down.

In this cohort CAG repeat lengths ranged from 37 to 62, with the majority (95%) of patients having repeats of less than or equal to 50. For each additional repeat, patients performed 5.6 fewer taps (p < 0.001; 95% CI = 8.0 to 3.3 fewer taps). Since age was included as a covariate in the model, it takes into account that individuals with the same CAG repeat length may be at different stages of the disease depending on their age. The number of taps at first visit as a function of CAG length, age, and sex can be seen in Figure 2. The two planes are parallel, with the female plane shifted downward by 33 taps, which represents the effect of sex (p = 0.0001). As can be seen, older patients and those with a larger number of repeats perform fewer taps. In addition, it can be seen that a male patient aged 20 years with 60 repeats performs a similar number of taps (approximately 150) as a male patient 75 years of age with 37 repeats. Next, we assessed whether patients with a greater number of CAG repeats progress at a faster rate by testing for a time by CAG length interaction. For each additional repeat, patients performed an additional 0.45 fewer taps every year (p = 0.008, 95% CI = 0.78 to 0.12 fewer taps), thus the rate of decline was faster for those with a greater number of repeats. This relationship can be seen in Figure 3.

### Change in hand tapping as a function of TFC/stage of disease

**Figure 4:**
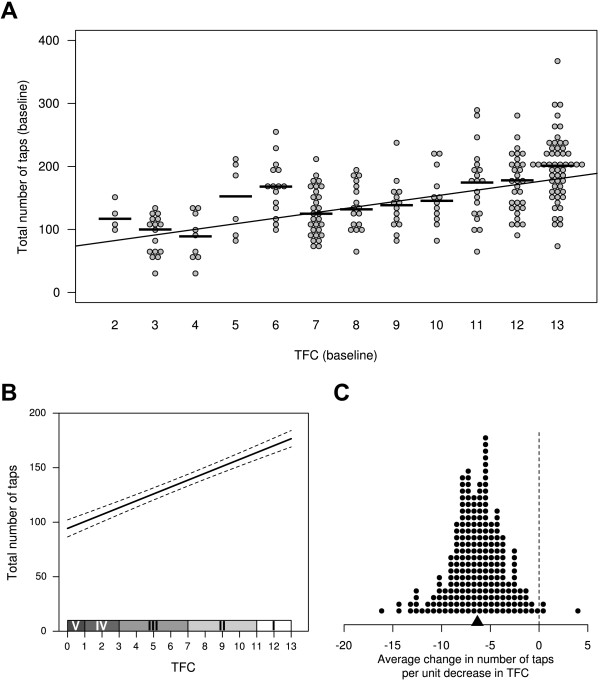
**Relationship between tapping and TFC.** Baseline cross-sectional analysis of the number of taps by TFC **(A)** for the 227 patients for which TFC data was available; horizontal lines indicate the median for each group. Population average for the association between tapping and TFC within patients with 95% CI **(B)**. On average, patients performed 6.3 fewer taps (triangle on the x-axis) for every unit decrease in TFC **(C)**.

It was also of interest to determine the relationship between the number of taps and TFC. This was done in two ways. First, a cross-sectional examination was made of patients using their baseline values (i.e. the first time they visited the clinic; Figure 4A). A linear model was used which included age and sex as covariates, and TFC as a continuous variable and found that for each unit decrease in TFC, the number of taps decreased by 8.4 (95% CI = 6.8 to 10.0; p < 0.0001). On its own, TFC accounted for 29% of the variance in the number of taps, and age and sex accounted for an additional 8%. The second analysis examined the relationship between tapping and TFC within individuals (Figure 4B and C), and in this case for each unit decrease in TFC, the number of taps decreased by 6.3 (95% CI = 5.4 to 7.1, p < 0.0001).

### Sample size estimates for a randomised trial

**Figure 5:**
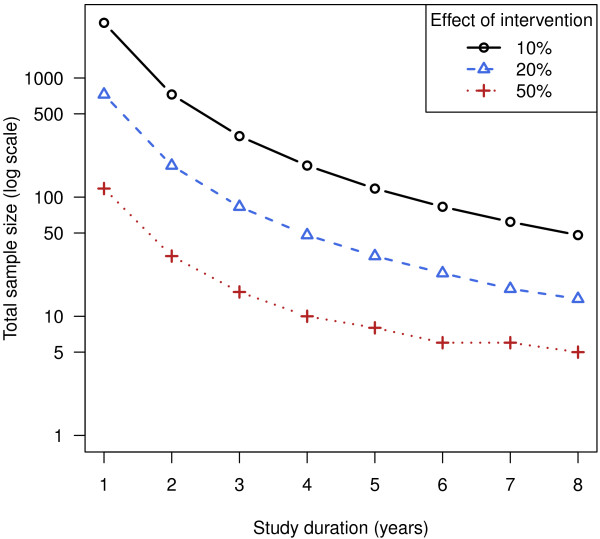
**Number of patients required for various effect sizes and follow-up times.** Since patients decrease (on average) at rate of 5.1 taps per year, a therapeutic intervention that results in a 20% improvement would have a rate of decline of 4.06 taps per year. For a two group experiment, this effect could be detected with 80% power after one year if 727 patients are recruited, or after four years with 48 patients. These estimates are approximate and depend on the proportion of individuals with long versus short CAG repeats, the number of patients lost to follow up, and the design of the study.

Based on the mean annual decrease of taps and the patient-to-patient variability in this analysis, it is possible to estimate the number of patients required for a randomized trial when using tapping as the primary outcome. Figure 5 displays the results for a two group (treated versus control) trial where the difference between groups is tested with a two-tailed independent samples t-test on the change in scores (number of taps at year *t* minus number of taps at year 0). Since the two groups will diverge over time if the treatment is effective, one can either increase the sample size or follow up the patients for a longer time to increase power. For the calculations, power = 80%, α = 0.05, and it is assumed that no patients are lost to follow-up or drop out. Since patients with a greater number of CAG repeats progress at a faster rate, the number of patients and/or follow up time can be reduced by enriching for this population.

## Discussion

This analysis used a simple motor hand tapping task to follow disease progression in HD patients, at all stages of the disease, to ascertain whether tapping changes reliably over time. We found that in this patient cohort the rate of voluntary hand tapping declined at a steady rate (linearly) of 5.1 taps per year on average (Figure 1C) and this rate of decline was similar between sexes. It extends our previous study in this area by looking at larger numbers of patients over longer time periods, on treatment, and thus has the advantage that it recruited all patients attending clinic and so represents real life practice.

Several studies have now demonstrated that simple, rather than complex, motor tasks are the most sensitive markers of disease onset and progression in HD, and include tasks such as hand and finger tapping [9,27,28], peg insertion task, grooved pegboard task [29], tongue force [5], and decision making reaction time [30]. The current study reinforces this point that a simple motor biomarker is useful for tracking disease course and that the impairment in voluntary movement captured in these patients using this approach is related to functional disability. We did not assess whether hand tapping would be a suitable surrogate biomarker for TFC or any other physiological endpoint such as striatal volume, nor whether tapping has any prognostic or predictive value. However, we have previously shown that changes in hand tapping over time correlates with UHDRS motor scores [18] and thus the number of taps could be used as a biomarker to assess the efficacy of compounds or other therapeutic interventions as it is noninvasive, inexpensive, and related to standard motor scores. In addition, tapping has an important advantage over "wet" biomarkers as it is not affected by sample quality, differences between labs or clinics in terms of sample preparation and handling, or differences between batches within a lab. It is therefore less likely to be affected by extraneous variables that can introduce bias and variability, making the analysis and interpretation of results more complex [31].

Although this study has clear advantages in terms of size and representativeness of the sample, there are a number of limitations. These include the lack of a control group and a paucity of other clinical or imaging measures with which to correlate our hand tapping data. As this is a retrospective study based on patients attending the clinic for their routine appointment, we did not include testing of controls as part of the study design. This would be useful to include in future studies to better understand the age dependent effects on the rate of hand tapping. The smaller number of patients with known CAG repeat lengths also reflects the fact that the study is retrospective and that many patients were diagnosed at a time when these were not routinely recorded as part of normal clinical practice. Finally, it should be realised that this task can also be affected by mood and fatigue, subject to a practice effect, and can be impaired in patients with orthopaedic or rheumatological problems [12]. We did not look specifically at any of these factors but over long time scales any practice effect would likely be negligible. In addition, the device does not rely on pressure sensitive taps or large movements, and therefore joint problems are unlikely to play a major part in determining the hand tap score.

## Conclusions

We have previously reported that hand tapping is a useful marker of motor dysfunction in HD [18] and this new longitudinal analysis with 237 patients confirms this initial work and extends the findings to a larger group of manifest patients, with a wider range of disease stages, and followed up for longer times. As such, we provide further evidence that this test reliably tracks disease progression and could therefore easily be adopted in clinical trials.

## Competing interests

The authors have no competing interests to disclose.

## Authors' contributions

RAB planned the analysis and collected the data. SEL performed the statistical analysis and interpretation. LMC helped collect and analyse the patient data. All authors contributed to writing the manuscript and read and approved the final version.

## Pre-publication history

The pre-publication history for this paper can be accessed here:

http://www.biomedcentral.com/1471-2377/14/35/prepub

## References

[B1] MacDonald ME Ambrose CM Duyao MP Myers RH Lin C Srinidhi L Barnes G Taylor SA James M Groot N MacFarlane H Jenkins B Anderson MA Wexler NS Gusella JF Bates GP Baxendale S Hummerich H Kirby S North M Youngman S Mott R Zehetner G SEDLACEK Z Poustka A Frischauf AM Lehrach H Buckler AJ Church D Doucettestamm L A novel gene containing a trinucleotide repeat that is expanded and unstable on huntingtons-disease chromosomes Cell 1993 72 971 983 10.1016/0092-8674(93)90585-E 8458085

[B2] Reiner A Albin RL Anderson KD D’Amato CJ Penney JB Young AB Differential loss of striatal projection neurons in huntington disease Proc Natl Acad Sci U S A 1988 85 5733 5737 10.1073/pnas.85.15.5733 2456581 PMC281835

[B3] Rosas HD Koroshetz WJ Chen YI Skeuse C Vangel M Cudkowicz ME Caplan K Marek K Seidman LJ Makris N Jenkins BG Goldstein JM Evidence for more widespread cerebral pathology in early HD: an MRI-based morphometric analysis Neurology 2003 60 1615 1620 10.1212/01.WNL.0000065888.88988.6E 12771251

[B4] Tabrizi SJ Langbehn DR Leavitt BR Roos RA Durr A Craufurd D Kennard C Hicks SL Fox NC Scahill RI Borowsky B Tobin AJ Rosas HD Johnson H Reilmann R Landwehrmeyer B Stout JC TRACK-HD Investigators Biological and clinical manifestations of huntington’s disease in the longitudinal TRACK-HD study: cross-sectional analysis of baseline data Lancet Neurol 2009 8 791 801 10.1016/S1474-4422(09)70170-X 19646924 PMC3725974

[B5] Tabrizi SJ Scahill RI Durr A Roos RA Leavitt BR Jones R Landwehrmeyer GB Fox NC Johnson H Hicks SL Kennard C Craufurd D Frost C Langbehn DR Reilmann R Stout JC TRACK-HD Investigators Biological and clinical changes in premanifest and early stage huntington’s disease in the TRACK-HD study: the 12-month longitudinal analysis Lancet Neurol 2011 10 31 42 10.1016/S1474-4422(10)70276-3 21130037

[B6] Reilmann R Kirsten F Quinn L Henningsen H Marder K Gordon AM Objective assessment of progression in huntington’s disease: a 3-year follow-up study Neurology 2001 57 920 924 10.1212/WNL.57.5.920 11552034

[B7] Beste C Stock AK Ness V Hoffmann R Lukas C A novel cognitive-neurophysiological state biomarker in premanifest huntington’s disease validated on longitudinal data Sci Reports 2013 3 1797 10.1038/srep01797 PMC3647202 23652721

[B8] Stout JC Jones R Labuschagne I O’Regan AM Say MJ Dumas EM Queller S Justo D Santos RD Coleman A Hart EP Durr A Leavitt BR Roos RA Langbehn DR Tabrizi SJ Frost C Evaluation of longitudinal 12 and 24 month cognitive outcomes in premanifest and early huntington’s disease J Neurol Neurosurg Psychiatry 2012 83 687 694 10.1136/jnnp-2011-301940 22566599 PMC3368487

[B9] Tabrizi SJ Reilmann R Roos RAC Durr A Leavitt B Owen G Jones R Johnson H Craufurd D Hicks SL Kennard C Landwehrmeyer B Stout JC Borowsky B Scahill RI Frost C Langbehn DR TRACK-HD Investigators Potential endpoints for clinical trials in premanifest and early huntington’s disease in the TRACK-HD study: analysis of 24 month observational data Lancet Neurol 2012 11 42 53 10.1016/S1474-4422(11)70263-0 22137354

[B10] Clabough E Huntington’s disease: the past, present, and future search for disease modifiers Yale J Biol Med 2013 86 217 233 23766742 PMC3670441

[B11] Kremer H Unified huntington’s disease rating scale: reliability and consistency Mov Disord 1996 11 136 142 8684382 10.1002/mds.870110204

[B12] Weir DW Sturrock A Leavitt BR Development of biomarkers for huntington’s disease Lancet Neurol 2011 10 573 590 10.1016/S1474-4422(11)70070-9 21601164

[B13] Rupp J Blekher T Jackson J Beristain X Progression in prediagnostic huntington disease J Neurol 2010 81 379 384 10.1136/jnnp.2009.176982 PMC2872788 19726414

[B14] Thompson JC Poliakoff E Sollom AC Howard E Automaticity and attention in huntington’s disease: when two hands are not better than one Neuropsychologia 2010 48 171 178 10.1016/j.neuropsychologia.2009.09.002 19747497

[B15] Paulsen JS Langbehn DR Stout JC Aylward E Ross CA Nance M Guttman M Johnson S MacDonald M Beglinger LJ Duff K Kayson E Biglan K Shoulson I Oakes D Hayden M Predict-HD Investigators and Coordinators of the Huntington Study Group Detection of huntington’s disease decades before diagnosis: the predict-HD study J Neurol Neurosurg Psychiatry 2008 79 874 880 10.1136/jnnp.2007.128728 18096682 PMC2569211

[B16] Tabrizi SJ Scahill RI Owen G Durr A Leavitt BR Roos RA Borowsky B Landwehrmeyer B Frost C Johnson H Craufurd D Reilmann R Stout JC Langbehn DR TRACK-HD Investigators Predictors of phenotypic progression and disease onset in premanifest and early-stage huntington’s disease in the TRACK-HD study: analysis of 36-month observational data Lancet Neurol 2013 12 637 649 10.1016/S1474-4422(13)70088-7 23664844

[B17] Rowe KC Paulsen JS Langbehn DR Duff K Self-paced timing detects and tracks change in prodromal huntington disease Neuropsychology 2010 24 435 442 20604618 10.1037/a0018905 PMC2900808

[B18] Michell AW Goodman AOG Silva AHD Lazic SE Morton AJ Barker RA Hand tapping: a simple, reproducible, objective marker of motor dysfunction in huntington’s disease J Neurol 2008 255 1145 1152 10.1007/s00415-008-0859-x 18465109

[B19] Blekher T Johnson SA Marshall J White K Hui S Saccades in presymptomatic and early stages of huntington disease Neurology 2006 67 394 399 10.1212/01.wnl.0000227890.87398.c1 16855205

[B20] Antoniades CA Xu Z Mason SL Carpenter RHS Barker RA Huntington’s disease: changes in saccades and hand-tapping over 3 years J Neurol 2010 257 1890 1898 10.1007/s00415-010-5632-2 20585954

[B21] Andrich J Saft C Ostholt N Müller T Complex movement behaviour and progression of huntington’s disease Neurosci Lett 2007 416 272 274 10.1016/j.neulet.2007.02.027 17321683

[B22] Saft C Andrich J Meisel NM Przuntek H Müller T Assessment of complex movements reflects dysfunction in huntington’s disease J Neurol 2003 250 1469 1474 10.1007/s00415-003-0256-4 14673581

[B23] Müller T Saft C Andrich J Harati A Diadochokinetic movements differ between patients with huntington’s disease and controls NeuroRehabilitation 2013 33 649 655 24018370 10.3233/NRE-130992

[B24] Goldberg YP Andrew SE Clarke LA Hayden MR A PCR method for accurate assessment of trinucleotide repeat expansion in huntington disease Hum Mol Genet 1993 6 635 636 8353482 10.1093/hmg/2.6.635

[B25] Shoulson I Fahn S Huntington disease clinical care and evaluation Neurology 1979 29 1 3 10.1212/WNL.29.1.1 154626

[B26] Shoulson I Huntington disease: functional capacities in patients treated with neuroleptic and antidepressant drugs Neurology 1981 31 1333 1335 10.1212/WNL.31.10.1333 6125919

[B27] Saft C Andrich J Meisel N-M Przuntek H Müller T Assessment of simple movements reflects impairment in huntington’s disease Mov Disord 2006 21 1208 1212 10.1002/mds.20939 16700032

[B28] Andrich J Saft C Ostholt N Müller T Assessment of simple movements and progression of huntington’s disease J Neurol Neurosurg Psychiatry 2007 78 405 407 17369593 10.1136/jnnp.2006.105338 PMC2077774

[B29] Maroof DA Gross AL Brandt J Modeling longitudinal change in motor and cognitive processing speed in presymptomatic huntington’s disease J Clin Exp Neuropsychol 2011 33 901 909 10.1080/13803395.2011.574606 21644140

[B30] Kirkwood SC Siemers E Bond C Conneally PM Christian JC Foroud T Confirmation of subtle motor changes among presymptomatic carriers of the huntington disease gene Arch Neurol 2000 57 1040 1044 10.1001/archneur.57.7.1040 10891987

[B31] Jenkins M Flynn A Smart T Harbron C Sabin T Ratnayake J Delmar P Herath A Jarvis P Matcham J PSI Biomarker Special Interest Group A statistician’s perspective on biomarkers in drug development Pharm Stat 2011 10 494 507 10.1002/pst.532 22162336

